# Indents

**Published:** 1917-10

**Authors:** 


### INDENTS

☞ **Brief Articles of Technical or Scientific Value will receive notice and comment. Contributions invited.**

*Sterilizing Root Canals* By drawing hot air into a chip blown from an alcohol flame and injecting the air thus heated we accomplish both dessicating by the heat and sterilization by the formaldehyde gas from the alcohol flame. By using a small and suitably shaped needle point the hot alcohol vapor can be injected into the entire length of the canal. Care should be exercised to prevent the injection of condensed steam which may accumulate in the syringe point.—Dr. Almon in *Review*.

*"Eat Wisely"* There is no dearth of advice about "what to eat" or "how to cut down the meat bill" or "foods we ought to know" or similar themes in these stirring days of rising costs of living. Some of the instructions are formulated in terms of menus which only a skilled housewife can easily interpret; others are expressed in the increasingly more popular language of calories with its implication of energy and consequent strength; still others abound in the platitudes of the food faker who has his "daily column" to be filled. We have rarely seen a more specific, sane and clearly understandable propaganda than that recently formulated by the Bureau of Home Economics of the New York Association for Improving the Condition of the Poor. In a leaflet aimed to suggest such meals as will be best for growing children, an expert's advice is summarized under this caption: To get the best results, spend money for food as follows:

1. Spend from one-fourth to one-third of your food money for bread, cereals, macaroni and rice.

2. Buy at least from a third to half a quart of milk a day for each member of the family.

3. Spend as much for vegetables and fruits together as you do for milk. If you use half a quart of milk for each member of the family, this may not always be possible. Then spend as much for vegetables and fruit as a third of a quart of milk a day would amount to.

4. Spend not more for meat and eggs than for vegetables and fruits. Meat and eggs may be decreased with less harm than any of the other foods mentioned. The amount spent for meat may decrease as the amount spent for milk increases.

*The Journal* concurs in these recommendations.—*Journal A. M. A.*, July 14, 1917.

*Packing Rubber to Make Tight Joints against the Teeth*

I have over thirty years' experience in this work, and if anyone thinks he can make tight joints by "flasking and packing red rubber," he is mistaken, it can't be done that way. But there is a method that I have used for many years that makes as tight a joint as is possible with rubber. If anyone will notice, the pink rubber around the tops of the teeth is always tight, the solution of the matter is to carry the pink rubber around the teeth, but not the pins. How many rubber workers know that pink rubber neither expands nor contracts in vulcanizing? Pink stays where it is put if the packing is carefully done, which in some cases requires considerable time. I make a solution of pink rubber in chloroform and before heating the flask for packing, paint the plaster where I want the pink rubber to stay. It is as easy as filling roots of teeth.—E. B. Davis in *Digest*.

*Insures His Teeth*

It is interesting to know that Johnny Hines, moving-picture actor appearing in films with Marie Dressler, has his teeth insured against loss by accidents, fights, etc. He knows that his grin is one of his chief assets and values his teeth to the extent that he is willing to pay a large premium for their insurance.—*Exchange.*

*A Protest* The man who has mastered the principles of a profession has a right to consider himself of superior mental attainment. Not in an egotistical and vainglorious way, but as a fact. Neither tradesman nor the business man, so called, has reached his mental standard. This is the principle difference between a business man and a man of the professions. A professional man has nothing to sell except his services. This is too personally intimate an asset to advertise without boasting. Boasting is bad form. No gentleman will do it.

A professional man's reward comes in fees, social prominence and distinction in the community, and the personal satisfaction in good service given, and the latter is not small to the right thinking man.

A man needs little education to be a business man. He can sell groceries even if he can not read. The same is true with a tradesman. A man may be a most excellent machinist and be illiterate, but if ambition spurs him to educate himself he learns mathematics, physics, chemistry, drawing and other collateral branches and becomes, not a better machinist, except incidentally, but a mechanical engineer—a Professional Man. So it is with the business man. Business may be made a profession, but not by the ignorant. Now this continual harping upon business in Dentistry by a few men who are themselves professional failures and generally business failures also, is crass impudence. The criticising of older successful ethical gentlemen who have established themselves in the hearts of all right-thinking members of the dental profession should be met by such universal and unmistakable rebuff as to put a stop to the growing evil of commercialism in our ranks. A commercialism which is only corrupting and is fostered and designed for the benefit and relief of the unprepared and inefficient. Men whose God is the dollar sign and who would drag down all ideals, suppress all sentiment, smother all poesy, rob life of all its sweetness, and do not conserve for the temporal needs of life, but for avarice and greed.—Editorial in July *Western Dental Journal.*

*Absorption of the Roots of the First Teeth* In examining large numbers of "milk teeth," Luciani noticed that the normal absorption of the roots occurred completely only when the pulp of the tooth was in normal condition. The physiologic integrity of the tooth is indispensable, he declares, in preparation for the normal process of second dentition. All his evidence proclaims the importance of preserving the vitality of the pulp of the first teeth until they are ready to drop off from absorption of their roots.—Luciani, in *Bulletin*, Paris Academy of Medicine.

*Pulp Devitalization* So thoroughly am I convinced of the inadequacy of pulp devitalization and of root filling as a proper therapeutic measure, that I am going to violate traditions and make a prophecy for you. I predict for you that in twenty-five years the man who wantonly destroys pulps of teeth will be held to as strict an accountability by the law as is the medical practitioner at the present time who fails to observe common, ordinary prudence in the treatment of a case.—F. B. Kremer, *Xi Psi Phi Quarterly.—Cosmos.*

*Infiltration Anesthesia* The infiltration method consists in inserting the one-inch needle over the root of tooth midway between gum line and apex, going under the periosteum until opposite the apex, and deposit from one-quarter to one c.c. of solution, being careful not to balloon the tissues, as the anesthesia is obtained by getting the absorption of the solution through the canaliculi of the bone to the nerve under the periosteum, and the smallest amount of solution (sometimes three minims will do it) is all that is necessary, if the technic is correct. Infiltration anesthesia can be used on ten anterior teeth of upper jaw and centrals and laterals in lower jaw successfully; the others are more or less doubtful as to results.—*Dental Summary.*

*Refining Casting Gold.* Never use the same gold for a second casting without cleaning. This is easily done by placing the gold on a charcoal block and fusing, and while the gold is kept in a fused condition a flux made of borax, one part, and potassium nitrate, three parts, is added a little at a time until all impurities have been removed. Gold so treated for a few minutes will insure a good casting.—Dr. W. D. N. Moon, in *Dental Review.*

*Pathologic Root Ends* Some years ago I began to study the question of teeth, and why it was that you had so much trouble in root canal fillings. I had never seen any roentgenograms of extracted teeth, so I took teeth which had been extracted and X-rayed them, and studied them with the magnifying glass. That proved very interesting.

Here are the plates, so there can be no question about the number of teeth. There were three hundred odd roentgenographed after extraction. I studied, I think, 318 of them. Those terminal canal apices are so small that with your naked eye you can not see the foramina; but take a magnifying glass and examine them, and you will find many of the most interesting conditions—things pointed out by Stein and others on the anatomy, and by Noyes on the histology, but never shown except with the X-rays.

In 318 teeth there were 516 roots that I defy any man, no matter how skillful he may be, to have filled to the very end.

Sixty-three cases were found to have multiple foramina.

The root canal ended at a sharp angle in 101 specimens.

The root canals were so curved that it would have been impossible to pass any instrument through in 53 cases.

Two canals coalesced and ended in one foramen in 70 specimens.

There were four or more roots in seven teeth.

Two root canals joined and then ended in multiple foramina in four teeth.

There were five bicuspids with very deep single pulp chambers, which ended in multiple short canals less than a sixth of an inch in length.

The root canals could not be defined in 165 specimens.

Evidence of marked root absorption at the apex was found in 96 specimens,

In no root was a complete root canal filling found.—Dr. W. H. Haskins, in *Journal of Allied Societies*.

*Duty of Membership*

Every member ought to feel a personal responsibility for the success of his society meetings and be willing to contribute whatever he can. He should strive to perfect some line of work so that he could give a clinic at any time on short notice if need be. Many men do not like to appear publicly to read papers or clinic, but none the less they should do their parts. Had it not been for the great numbers of unselfish men who have made dentistry free and open to all by their generous cooperation and giving of their time and discoveries, we should never have made the progress that we have or been a profession at all. The time now is when everyone should give out all he knows. We owe it to our traditions and the good of all mankind to strive to give up all that we can for our own development and the good of humanity.—Dr. E. A. Bonar, in *Western Journal*.

*The Open Door*

One is quite apt to hear much said, and can read much that is written upon the democracy of science. We are told, and rightly so, that science recognizes no geographic boundaries, that the searcher for truth is glad to find it in the workshops of either friend or foe.

Such a premise as the above can be rightfully applied to the genuine seeker for knowledge, but to the poser and the pseudoscientist this statement has little meaning. Truth is the common property of all, and he who seeks to hide its light from the world for personal gain is an enemy to progress and a menace to the cause he has espoused.

It is said of liberty that many crimes have been committed in its name. The same is equally true of science. Behold the self-appointed gods in some of the many fields of human endeavor, blowing the trumpet of accomplishment, and expecting to see the servient knee of all its devotees bend to the ground; and unless the bending is in evidence, the fires of wrath are kindled and the unholy ones must be cast out into eternal darkness where, figuratively speaking, there is weeping and gnashing of teeth.

Such an attitude on the part of men is cause enough to elicit genuine pity for them—pity that they are so far away from the goal they hope to reach, and pity that they can do damage to some with whom they are thrown in contact. Men of this type are known to all, and, unfortunately, they grace—more correctly speaking, disgrace—both the dental and the medical profession. The "holier than thou" attitude is assumed by them, and he who has the temerity to disagree with their conclusions in the realm of science is in danger of being crushed. This attitude is wrong, and no genuine scientist ever assumes it. Truth is big enough to encompass all. The man who genuinely seeks it has no time to waste in condemning the efforts of his fellow workers.

There is plenty of room in the realm of science for all, but there is no place in this realm for the individual who wants to usurp authority, decry the efforts of his coworkers and stigmatize the results that others have secured through arduous toil.

There is too much to accomplish in dentistry and medicine to waste one's time and energy trying to placate, understand or affiliate with the type that we have described. Like the biblical poor, they will always be with us. Let them bay at the moon, while the tried and genuine disciples of truth continue in their endeavor to open wider and still wider the door.—*International Journal of Orthodontia.*

*Protecting Patients* Should caries of the teeth occur it is the duty of the dentist to prevent its effects from endangering the health of his patient. This he may do in one or all of four ways:

1. By endeavoring to keep the pulp vital. Failing in this,
2. He should scientifically treat the root canals. Failing in this,
3. He may resort to root apex amputation. Failing in this,
4. He should advise extraction.—B. B. Palmer, Jr., in *Journal of Allied Societies*, March, 1917.

